# CD20-Positive B-Cell Non-Hodgkin Lymphoma Associated With Recurrent Acute Pancreatitis, Bilateral Renal Lesions, and Retroperitoneal Lymphadenopathy

**DOI:** 10.7759/cureus.116919

**Published:** 2026-09-25

**Authors:** Rehan Sarfraz, Magnie L Ledesma Leon, Dunia Nabi, Laura Hernandez Carratala, Jennifer Fuller

**Affiliations:** 1 Internal Medicine, Palmetto General Hospital, Miami, USA; 2 Internal Medicine, American University of the Caribbean School of Medicine, Miami, USA

**Keywords:** acute kidney injury (aki), bilateral renal infiltration, cd20-positive b-cell non-hodgkin lymphoma, diffuse large b-cell lymphoma (dlbcl), lymphoma with pancreas infiltration, lymphoma with renal infiltration, recurrent acute pancreatitis

## Abstract

Acute pancreatitis is most often caused by gallstones, alcohol use, or hypertriglyceridemia, whereas malignancy is an uncommon association. We report a case of a 29-year-old woman with recurrent acute pancreatitis without a clearly dominant, identifiable cause. Although she reported intermittent heavy weekend alcohol use, recurrence after initial improvement, increasing lactate dehydrogenase (LDH), bilateral renal cortical lesions, and progressive retroperitoneal lymphadenopathy suggested a systemic process. Core biopsy of a retroperitoneal lymph node demonstrated a CD20-positive B-cell non-Hodgkin lymphoma with a Ki-67 proliferation index of up to 80%. Limited tissue and nondiagnostic flow cytometry prevented definitive subclassification; diffuse large B-cell lymphoma (DLBCL) remained in the differential diagnosis. One documented cycle of rituximab, cyclophosphamide, doxorubicin, vincristine, and prednisone (R-CHOP) was administered during a subsequent hospitalization, with short-term symptomatic, pancreatic enzyme, and renal improvement. The patient was subsequently lost to follow-up. This case emphasizes the importance of considering lymphoproliferative disease when recurrent pancreatitis is accompanied by progressive systemic imaging abnormalities.

## Introduction

Acute pancreatitis is a common condition encountered in clinical practice, most frequently attributed to gallstones and alcohol use. Other causes, including hypertriglyceridemia, medications, and autoimmune disease, are less common but still well recognized. In most cases, the underlying etiology can be identified with a careful history, laboratory evaluation, and imaging [1,2].

Malignancy is a rare but important cause of pancreatitis and may occur through pancreatic infiltration, ductal obstruction, or external compression from adjacent structures [3,4]. While pancreatic adenocarcinoma is more commonly associated with pancreatitis, lymphoma involving the pancreas is uncommon and often not considered early in the diagnostic process [3-5].

B-cell non-Hodgkin lymphomas comprise a heterogeneous group that includes aggressive subtypes, such as diffuse large B-cell lymphoma (DLBCL), which frequently involves extranodal sites including the gastrointestinal tract and kidneys [6,7]. Pancreatic involvement by lymphoma is uncommon, and presentation in association with acute pancreatitis is especially rare [3,5,8]. This report describes a CD20-positive B-cell non-Hodgkin lymphoma detected during evaluation of recurrent acute pancreatitis and highlights the clinical, laboratory, and imaging clues that prompted tissue diagnosis.

## Case presentation

A 29-year-old woman with a history of pyelonephritis presented with five days of epigastric pain radiating to the back, associated with nausea, vomiting, and diarrhea. The pain worsened after meals and was partially relieved with nonsteroidal anti-inflammatory medications. She reported intermittent heavy alcohol consumption on weekends during the preceding months but denied daily or chronic use. Alcohol therefore remained a possible contributing factor rather than being categorically excluded.

On arrival, she was hemodynamically stable. The physical examination was notable for epigastric tenderness without guarding or rebound. Laboratory evaluation demonstrated a markedly elevated lipase level of 7,704 U/L and mild renal dysfunction, with a creatinine level of 1.1 mg/dL and an estimated glomerular filtration rate (eGFR) of 65 mL/min/1.73 m². Triglycerides were mildly elevated at 203 mg/dL, while calcium and liver biochemical tests were within reference limits: calcium 9.3 mg/dL, aspartate aminotransferase (AST) 28 U/L, alanine aminotransferase (ALT) 14 U/L, alkaline phosphatase (ALP) 103 U/L, and total bilirubin 0.9 mg/dL. Lactate dehydrogenase (LDH) was mildly elevated at 253 U/L.

Right upper quadrant ultrasonography showed no gallstones or biliary obstruction; the common bile duct measured 0.5 cm. Contrast-enhanced computed tomography (CT) of the abdomen and pelvis showed no significant pancreatic abnormality. It demonstrated multifocal, poorly marginated, predominantly cortical, wedge-shaped hypoenhancing lesions in both kidneys without mass effect, a discrete enhancing renal mass, hydronephrosis, or renal abscess (Figure 1). The initial radiologic differential included multifocal pyelonephritis, renal infarction, and an infiltrative process [9,10]. 

**Figure 1:**
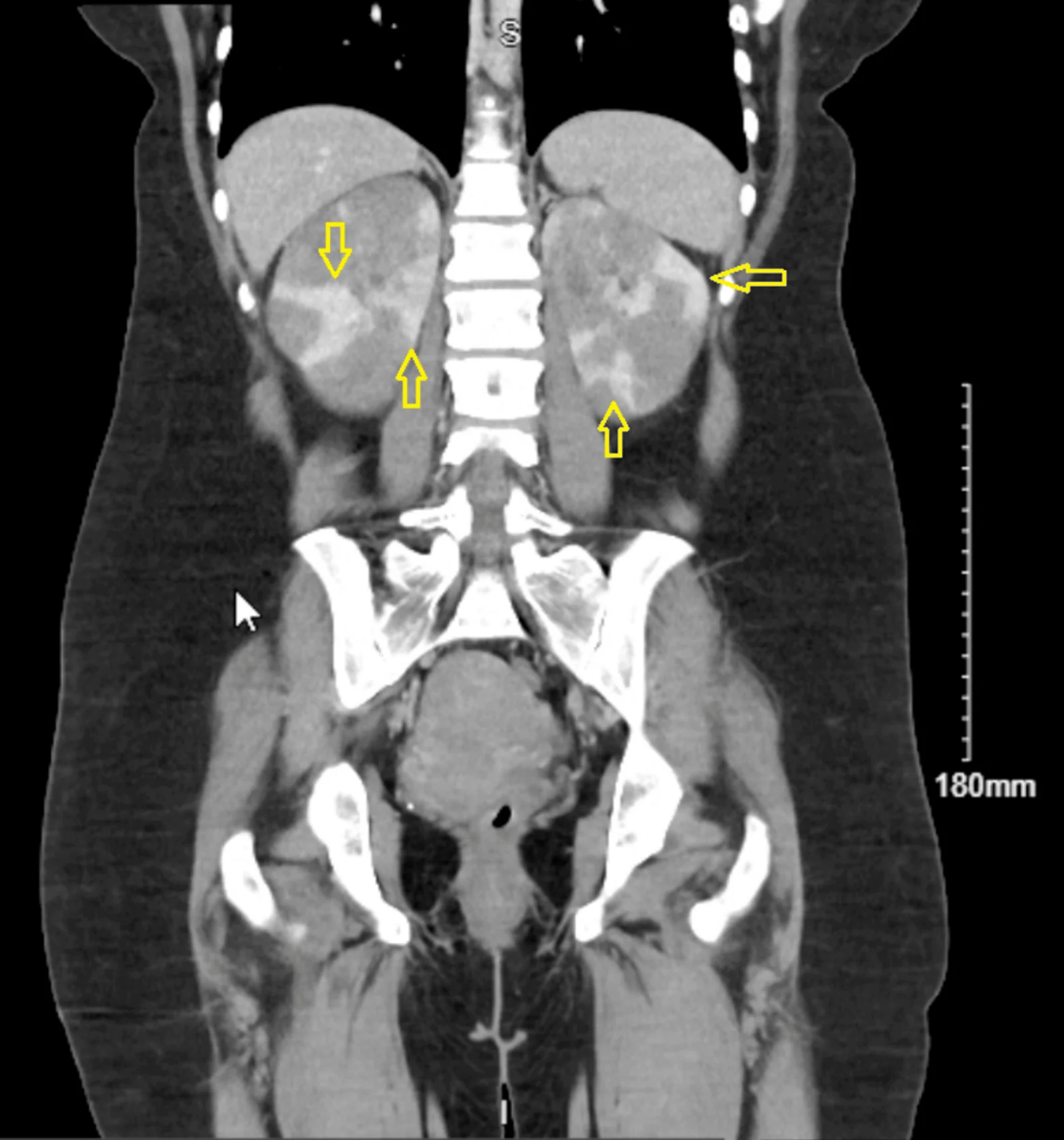
Contrast-enhanced CT abdomen demonstrating bilateral renal cortical hypoenhancing lesions, concerning for renal involvement by an infiltrative process such as lymphoma. Arrows identify representative renal cortical lesions. CT, computed tomography.

The patient was treated with intravenous fluids, bowel rest, and analgesia, with initial symptomatic improvement. She returned four days after discharge with recurrent epigastric pain. She denied fever, night sweats, and unintentional weight loss. Lipase was 1,725 U/L, while renal function had worsened to a creatinine level of 1.6 mg/dL and an eGFR of 44 mL/min/1.73 m². Triglycerides were 202 mg/dL, calcium was 8.8 mg/dL, and liver biochemical tests remained within reference limits. LDH increased to 650 U/L. Selected laboratory findings from both hospitalizations are summarized in Table 1.

**Table 1:** Selected laboratory findings during the first and second hospitalizations. Values are selected results from each hospitalization. Lipase, creatinine, eGFR, calcium, liver biochemical tests, and complete blood count values reflect presentation; triglyceride and LDH values represent measurements obtained during the respective hospitalization. ALP, alkaline phosphatase; ALT, alanine aminotransferase; AST, aspartate aminotransferase; eGFR, estimated glomerular filtration rate; LDH, lactate dehydrogenase; WBC, white blood cell count.

Laboratory value	First admission	Second admission	Reference range
Lipase, U/L	7,704	1,725	23-300
Creatinine, mg/dL	1.1	1.6	0.52-1.04
eGFR, mL/min/1.73 m²	65	44	≥90
Triglycerides, mg/dL	203	202	40-150
Calcium, mg/dL	9.3	8.8	8.4-10.2
AST, U/L	28	29	14-36
ALT, U/L	14	13	9-52
ALP, U/L	103	83	38-126
Total bilirubin, mg/dL	0.9	0.30	0.20-1.30
LDH, U/L	253	650	120-246
WBC, ×10³/µL	8.3	7.9	5.0-11.0
Hemoglobin, g/dL	13.3	12.1	12.0-16.0
Platelets, ×10³/µL	311	223	130-450

Evaluation for alternative causes included a negative urine drug screen and an unrevealing autoimmune assessment. The immunoglobulin G (IgG) subclass panel was within reference limits, including an IgG4 concentration of 11 mg/dL, and the antinuclear antibody screen was nonreactive. She denied use of glucagon-like peptide-1 receptor agonists. Magnetic resonance cholangiopancreatography (MRCP) and endoscopic ultrasonography were considered but were not performed during the first two hospitalizations.

Repeat CT demonstrated interval progression of the retroperitoneal lymphadenopathy together with persistent bilateral renal cortical lesions, increasing concern for a systemic lymphoproliferative process (Figure 2).

**Figure 2:**
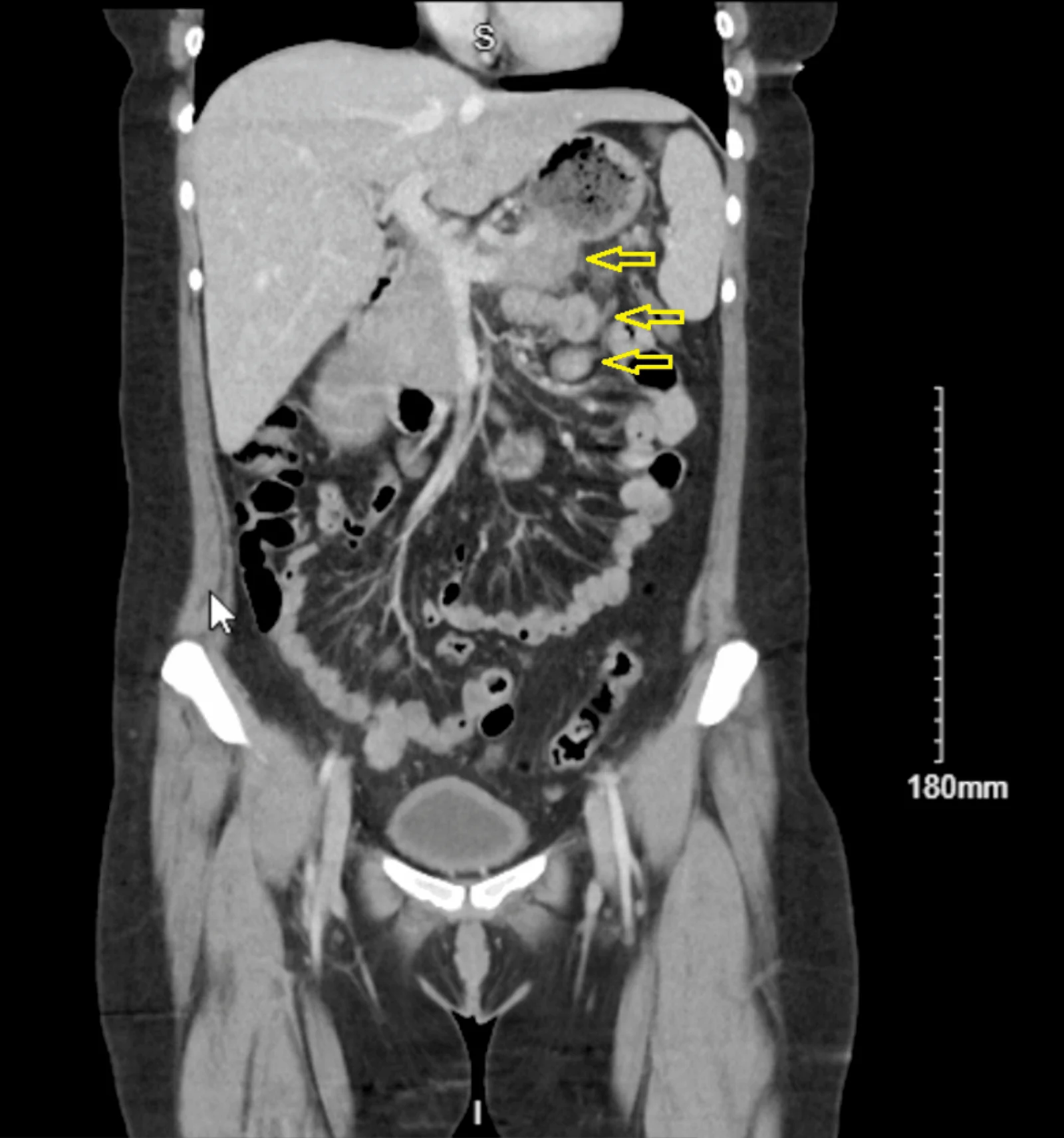
Contrast-enhanced CT of the abdomen and pelvis demonstrating progressive retroperitoneal lymphadenopathy. Arrows identify the enlarged retroperitoneal lymph nodes. CT, computed tomography.

Because no single common etiology fully explained the recurrent pancreatitis and imaging showed progressive lymphadenopathy with suspected extranodal disease, hematology/oncology was consulted. Fine-needle aspiration and core-needle biopsy of a retroperitoneal lymph node were performed. Neither pancreatic nor renal tissue was sampled.

The retroperitoneal lymph node core biopsy demonstrated an atypical lymphoid infiltrate morphologically and immunophenotypically compatible with a CD20-positive B-cell non-Hodgkin lymphoma with a high Ki-67 proliferation index of up to 80%. The atypical cells were strongly and diffusely positive for CD20, PAX5, and BCL6; focally positive for CD23 and BCL2; and negative for CD5, CD10, cyclin D1, CD30, and CD138. Pancytokeratin staining was negative for carcinoma. Flow cytometry was nondiagnostic because the specimen had low cellular yield and poor viability.

B-cell lymphoma fluorescence in situ hybridization (FISH) demonstrated no identified abnormalities; however, testing for BCL6 rearrangement was suboptimal and inconclusive. The limited core contained both smaller lymphoid cells and focal higher-grade areas with medium-to-large atypical cells. The differential included a CD5-negative/CD10-negative B-cell lymphoma, CD10-negative follicular lymphoma, and partial sampling of a higher-grade lymphoma such as DLBCL or double-/triple-hit lymphoma. An excisional lymph node biopsy was recommended for definitive subclassification but was not documented.

Formal Ann Arbor staging, positron emission tomography-computed tomography (PET-CT), bone marrow biopsy results, and an International Prognostic Index score were not available during the reported course; therefore, a retrospective stage was not assigned [11,12].

During a subsequent hospitalization, one cycle of rituximab, cyclophosphamide, doxorubicin, vincristine, and prednisone (R-CHOP) was administered and was tolerated without an immediate complication. Approximately five weeks after the initial presentation, the patient reported resolution of abdominal pain, nausea, and vomiting before discharge. Lipase decreased from 2,628 U/L to 458 U/L, and creatinine improved from 2.0 mg/dL to 1.6 mg/dL. No renal-specific chemotherapy dose modification was documented. Subsequent treatment cycles, interim restaging, and survival status were unavailable because the patient was lost to follow-up.

The patient provided verbal informed consent for publication of this case report and the accompanying radiologic images.

## Discussion

This case describes a CD20-positive B-cell non-Hodgkin lymphoma identified during evaluation of recurrent acute pancreatitis. Although gallstones, alcohol use, hypertriglyceridemia, medications, and autoimmune disease account for many pancreatitis cases, recurrence without a clearly dominant cause should prompt reconsideration of the differential, particularly when systemic abnormalities emerge [2,13].

Lymphoma involving or affecting the pancreas is rare and may be difficult to recognize early. Symptoms can resemble common gastrointestinal disorders, delaying recognition until lymphadenopathy or other extranodal findings become evident [3-5,8,14].

B-cell non-Hodgkin lymphomas can involve extranodal sites, including the kidneys [6,7]. In this patient, progressive retroperitoneal lymphadenopathy and bilateral renal cortical lesions supported a systemic process rather than isolated pancreatic inflammation. Renal lymphoma has variable imaging appearances and may resemble pyelonephritis or renal infarction [9,10]. The subsequent improvement in renal function after immunochemotherapy supported but did not histologically prove renal involvement.

Several mechanisms may explain an association between lymphoma and pancreatitis, including direct pancreatic infiltration, pancreatic duct obstruction, or compression by adjacent lymphadenopathy [3,5,15]. However, the initial CT showed no definite pancreatic lesion, and pancreatic tissue was not biopsied. Therefore, pancreatic infiltration and a direct causal relationship between the lymphoma and pancreatitis could not be confirmed and represent an important limitation of this report.

Intermittent heavy weekend alcohol use was a potential confounding factor and could not be excluded as contributory. Nevertheless, the patient denied daily or chronic use, and the recurrent episode occurred together with progressive systemic imaging abnormalities. Triglyceride concentrations near 200 mg/dL represented only mild elevation and were well below concentrations typically associated with hypertriglyceridemia-induced pancreatitis. Calcium and initial liver biochemical tests were normal; gallbladder ultrasonography showed no stones or obstruction; urine drug screening was negative; and testing for autoimmune pancreatitis was unrevealing, including an IgG4 concentration of 11 mg/dL and a nonreactive antinuclear antibody screen. Medication review did not identify glucagon-like peptide-1 receptor agonist use. MRCP and endoscopic ultrasonography were not performed during the first two hospitalizations.

The increasing LDH provided an additional clue. Although nonspecific, LDH is commonly elevated in aggressive lymphomas and may reflect cellular turnover and tumor burden. It is also incorporated into prognostic scoring systems for DLBCL [7].

The absence of classic B symptoms does not exclude lymphoma [7]. Formal Ann Arbor staging, PET-CT, bone marrow biopsy results, and an International Prognostic Index score were not available, so a retrospective stage was not assigned. This limits assessment of disease extent and prognosis.

Tissue biopsy remains essential for diagnosing lymphoma [6,7]. In this case, the limited core biopsy supported a CD20-positive B-cell lymphoma with a high proliferative index but did not permit definitive subclassification. DLBCL and other higher-grade B-cell lymphomas remained in the pathologic differential. R-CHOP is a standard first-line regimen for DLBCL [16], and one cycle was administered based on the treating hematology team's clinical assessment. Individual doses and renal-specific dose modifications were not documented in the available records.

This case underscores the importance of maintaining a broad differential when evaluating recurrent pancreatitis [2,13]. Published reports have similarly described DLBCL and other lymphomas presenting in association with acute pancreatitis [3,8,17]. Progressive lymphadenopathy and extranodal imaging abnormalities should prompt timely hematologic evaluation and tissue sampling while avoiding an unsupported assumption of direct pancreatic infiltration.

## Conclusions

B-cell non-Hodgkin lymphoma may present in association with recurrent acute pancreatitis. In this patient, progressive retroperitoneal lymphadenopathy, bilateral renal lesions, increasing LDH, and recurrence after initial improvement were important diagnostic clues. Retroperitoneal lymph node biopsy established a CD20-positive B-cell lymphoma with a high proliferative index, although limited tissue prevented definitive subclassification and pancreatic involvement was not histologically confirmed. Clinicians should consider lymphoproliferative disease when recurrent pancreatitis accompanies systemic imaging abnormalities and pursue timely tissue diagnosis.
